# The Throat Hospital, Golden Square

**Published:** 1895-10-05

**Authors:** R. Courtenay Welch, Greville Macdonald


### Editor's Letter-Box.

[Our correspondents are reminded that prolixity is a great bar to publication, and that brevity of style and conciseness of statement greatly facilitate early insertion.]

### THE THROAT HOSPITAL, GOLDEN SQUARE.

Sir,—While we thought it expedient to announce publicly the severance of our connection with the Throat Hospital, we did not think it necessary in the interests of the institution to state at the time our reasons for so doing. As, however, others think differently, we now ask you to publish the following statement.—We are, yours obediently,

Sutherland.
R. Courtenay Welch.
September 25th, 1895. Greville Macdonald.

Statement.

At the annual general meeting of the governors and subscribers of the Throat Hospital, held on April 10th last, after Mr. Courtenay Welch and two others of the retiring members had been unanimously re-elected, four other gentlemen (Dr. Norris Wolfenden, a former member of the honorary medical staff of the hospital, Major Probyn, Mr. Allom, and Mr. Osborne) were, after opposition, elected to serve on the committee of management. A few days later it was discovered that among those who had voted in support of these gentlemen were several persons who, in order to obtain a colourable qualification to vote under by-law 16 ("No annual subscriber shall be entitled to vote until after the payment of his subscription for the second year"), had, a few days before the meeting, paid to the secretary a subscription with the request that one moiety should be entered as a subscription for 1894 and the remainder as for 1895: one of the four gentlemen thus elected having himself in this manner obtained his qualification to vote. Although some of these payments were made just before the monthly committee meeting of April 2nd, the secretary made no mention to the committee of this unusual procedure; nor did he at the annual general meeting, when the qualification of one voter was challenged, give any hint that anything irregular had taken place.

At the next committee meeting held on May 7th Dr. Macdonald proposed, and Mr. Welch seconded, a resolution to the effect that a special general meeting of the governors and subscribers should be called to reconsider the opposed resolutions passed at the annual meeting. When this proposal was rejected, and the committee thus refused to allow the question to be reopened and the facts laid before the subscribers, Dr. Macdonald and Mr. Welch at once resigned.

The Duke of Sutherland, at the earnest request of Mr. G. H. Pember, the new chairman, and of Dr. Macdonald, who, acting independently of each other, pointed out the irreparable injury which his Grace's resignation would inflict upon the hospital, consented to defer any definite step until it could be ascertained whether the four gentlemen, the validity of whose election had been called in question, would voluntarily retire. A deputation from the committee subsequently waited upon the president; but as the arrangement he had suggested was not carried out, he had no alternative but to resign.

⁎⁎⁎ We need not point out the extreme gravity of the above statement, and can only repeat the advice we have already given, viz., that the subscribers should demand a complete investigation of allegations so definitely put forward. —Ed. *T. H.*

